# Ultrathin Quasi‐2D Perovskite‐Organic Segregated‐Structure Coupling for High‐Performance Narrowband Photomultiplication Detectors

**DOI:** 10.1002/advs.75123

**Published:** 2026-04-02

**Authors:** Hongfei Qu, Peng He, Xingchao Zhao, Yongchao Xie, Fulong Huang, Xiaoling Ma, Byung Hui Lee, Han Young Woo, Yumeng Shi, Zuliang Zhuo, Fujun Zhang, Xixiang Zhu

**Affiliations:** ^1^ Key Laboratory of Luminescence and Optical Information School of Physical Science and Engineering Ministry of Education Beijing Jiaotong University Beijing China; ^2^ Tangshan Research Institute of Beijing Jiaotong University Tangshan China; ^3^ Organic Optoelectronic Materials Laboratory Department of Chemistry College of Science Korea University Seoul Republic of Korea

**Keywords:** energy transfer, narrowband photodetectors, organic, photomultiplication, quasi‐2D perovskite

## Abstract

High spectral selectivity and low power operation remain a core goal for miniaturized optoelectronic sensors and imaging systems. In this work, a coupled separation structure based on quasi‐2D perovskite (BA_2_MA_4_Pb_5_I_16_) and a trap‐type organic bulk‐heterojunction (P3HT: PC_71_BM = 100:1, wt/wt) is employed to realize a highly spectrally selective photomultiplication‐type narrowband photodetector (PM‐NPD) with low‐power operation. The intrinsically ordered crystalline structure of the quasi‐2D perovskite layer suppresses directional energy transfer and promotes efficient hole tunneling injection from the adjacent organic photoresponse layer. Simultaneously, I/Br halogen gradient engineering is employed to precisely tune the bandgap, thereby enhancing optical performance and enabling selective detection at the target wavelength of 600 nm. The organic layer induces electron trapping and interfacial band bending, facilitating efficient hole tunneling injection at low bias. Consequently, the optimally designed PM‐NPDs with a segregated structure consist of a quasi‐2D perovskite layer coupled with an organic layer, achieving a peak EQE of 1200% and a D^*^ of 1.82 × 10^11^ Jones at a bias voltage of 3 V. Notably, the device incorporates a quasi‐2D perovskite layer with a thickness of 110 nm, which provides a critical foundation for the miniaturization and high‐density integration of portable and flexible optoelectronic devices.

## Graphical abstract

Quasi‐2D perovskite‐organic segregated‐structure coupling enables low‐power narrowband photomultiplication photodetectors with 110 nm perovskite layer. The optimal device achieves 1200% EQE and 1.82 × 10^1^ ^1^ Jones D^*^ at 3 V, offering a new strategy for miniaturized high‐selectivity optoelectronic sensing.

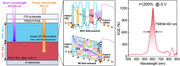

## 1 Introduction

Photodetectors (PDs) stand as core optoelectronic devices for converting optical signals into electrical signals, which are employed in high‐resolution imaging, optical communications, environmental monitoring, and biomedical sensing [1, 2, 3, 4]. However, amid portable electronics miniaturization and rising demand for energy‐efficient sensing, advanced PDs require high spectral selectivity for non‐target noise reduction and low‐voltage operation, which conventional narrowband PDs (NPDs) often fail to meet [5, 6, 7, 8]. Specifically, traditional NPDs rely on external optical filters such as interference filters and Bragg reflectors paired with broadband detectors, which increases device thickness to tens of micrometers and causes interface reflection losses that reduce photon efficiency [9, 10, 11]. Thin‐film NPDs based on intrinsic spectral narrowing mechanisms, such as charge collection narrowing (CCN), exciton dissociation narrowing (EDN), and charge injection narrowing (CIN), avoid external filters but face their own limitations. For instance, CCN‐based NPDs require thick active layers (>1 µm) to deplete short‐wavelength carriers, leading to slow response times and low responsivity [12, 13, 14]. CIN‐based photomultiplication (PM)‐NPDs achieve high gain but typically demand operating biases exceeding 10 V, which is incompatible with wearable power supplies [15, 16]. Even self‐driven perovskite NPDs, which utilize trap‐assisted carrier annihilation for narrowband response, suffer from weak photoresponse (EQE<10%) due to carrier loss in thick absorption layers [17]. These persistent trade‐offs between spectral selectivity, response speed, and power consumption have hindered the practical deployment of NPDs. Recent typical works on PM‐NPDs are summarized in Table S1. It is evident that most reported PM‐NPDs still rely on high operating biases (often exceeding −10 V) and complex device structures to trigger efficient PM, which poses a fundamental obstacle for low‐power integration and portable applications. This high‐voltage requirement fundamentally limits the compatibility of PM‐NPDs with emerging portable and wearable electronics, which rely on low‐voltage energy sources such as standard 3 V coin‐cell batteries (e.g., CR2032) [5]. The high operating bias of conventional PM‐NPDs far exceeds the output capability of such batteries. These issues cannot be effectively addressed through thick‐film approaches. These limitations collectively impede the practical application of narrowband photodetectors.

To address these limitations, hybrid architectures combining perovskite and organic semiconductors have emerged as a promising solution, namely tandem‐like or quasi‐tandem PDs. Perovskites exhibit exceptional optical and electrical properties, including high absorption coefficients (>10^5^ cm^−1^), tunable bandgaps via halide composition engineering, and compatible energy levels with organic materials [18, 19, 20, 21]. Organic semiconductors, by contrast, enable low‐cost solution processing, flexible device fabrication, and controllable carrier transport via bulk‐heterojunction (BHJ) design, which is critical for realizing PM at low biases [22, 23, 24]. The synergy between these two material systems addresses the inherent drawbacks of single‐component NPDs, with perovskites acting as wavelength‐selective filters to suppress background noise and organic heterojunctions serving as PM‐active layers to generate high photocurrent at low bias. As demonstrated by Zhao et al., quasi‐tandem perovskite/organic NPDs achieved sub‐microsecond response times and tunable narrowband response (full width at half maximum, FWHM = 41 nm) by utilizing the perovskite filtering ability and the organic layer carrier transport regulation [12]. Similarly, Zhao et al., reported perovskite/organic heterojunction NPDs with a spectral rejection ratio (SRR) of 1547 under bias light modulation, highlighting the potential of this hybrid design for high‐selectivity detection [25]. Within the perovskite family, 2D perovskites offer unique advantages over their 3D counterparts with their quantum‐well structure, tunable bandgap, and excellent environmental stability, effectively suppressing background noise and maintaining long‐term performance, making them ideal for integration into hybrid NPDs. Here, typical works on the NPD integration of perovskite and organic blend heterojunction photoresponse layers are summarized in Table 1.

**TABLE 1:** Performance of the typical perovskite‐organic heterostructure NPDs.

Layers	Perovskite Layer Thickness	λ peak [nm]	FWHM [nm]	Peak EQE [%]	Ref.
FAMAPbI_3_/PFN‐Br/PM6:Y6	N/A	780‐950	<100	80(@−1v) >100 (@540 nm and 0.1v)	[26]
MAPbI_3_/PFN/PM6:IT‐4F	750 nm	790	41	24	[12]
MAPbI_3_/PFN/PM6:Y6	550 nm	835	131	83.7	[27]
MAPbI_3_/PTB7‐Th:PC_71_BM:Y6	∼1000 nm	810	110	32.3	[28]
MAPbI_3_/PFN/PBQx‐TF:BTP‐eC9	1100 nm	750‐950	N/A	N/A	[25]
MAPbI_3_/CuSCN/PM6: Y6	1140 nm	810	95	74	[13]
MAPbI_3_/ PTB7‐Th: PC_71_BM	1700 nm	800	<50	N/A	[14]
MAPbI_3_/PTB7‐Th:COTIC‐4F	∼500 µm Single crystal	825	38	2259 (@−15v)	[7]
BA_2_MA_4_Pb_5_I_16‐x_Br_x_/P3HT:PC_71_BM	110 nm	600	60	1200 (@−3v)	**This work**

Despite advances in hybrid perovskite‐organic NPDs, persistent trade‐offs remain, such as high operating bias for PM thick active layers for spectral narrowing and low EQE for miniaturized devices. Herein, a series of PM‐NPDs based on a quasi‐2D perovskite‐organic coupled separation structure were designed to address these issues. The device architecture consists of two key layers: (1) a quasi‐2D perovskite (BA_2_MA_4_Pb_5_I_16_) layer fabricated with an ordered dispersion of nanoplates featuring varying n‐values with tunable bandgap via I/Br gradient engineering, which selectively transmits target wavelengths while suppressing the energy transfer from the top to the bottom surface; (2) an organic BHJ (P3HT: PC_71_BM, 100:1 wt/wt) as the PM‐active layer, where discontinuous electron transport channels induce electron trapping and interfacial band bending, enabling hole tunneling injection at low bias. Based on this design, we systematically investigate the effects of quasi‐2D perovskite thickness, deposition method, and halide composition on device performance. This work demonstrates the synergistic potential of quasi‐2D perovskite filtering and organic PM, providing a new strategy for the development of low‐power, high‐selectivity spectral detection with compatibility for miniaturization in PM‐NPDs.

## 2 Results and Discussion

**FIGURE 1:**
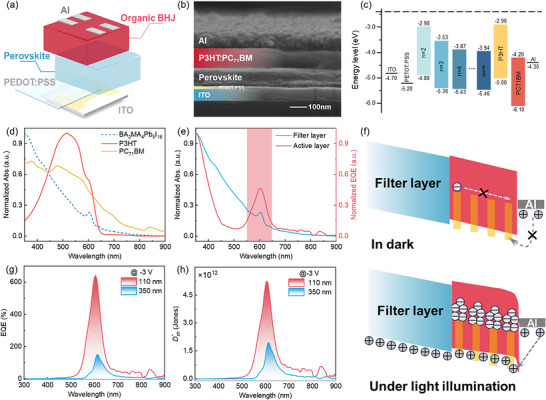
(a) Schematic structure diagram of quasi‐2D perovskite‐organic PM‐NPDs. (b) Cross‐sectional SEM image of quasi‐2D perovskite‐organic PM‐NPDs. (c) The energy levels of materials utilized in quasi‐2D perovskite‐organic PM‐NPDs. (d) The normalized absorption spectra of pristine quasi‐2D perovskite BA_2_MA_4_Pb_5_I_16_, P3HT, and PC_71_BM films. (e) The normalized absorption spectra of quasi‐2D perovskite layer and the normalized EQE spectra of the organic photoresponse layer. (f) The working mechanism of the PM organic layer in the quasi‐2D perovskite‐organic PM‐NPDs. Quasi‐2D perovskite PM‐NPDs based quasi‐2D perovskite films with thickness of 110 and 350 nm (g) EQE spectra measured at −3 V; (h) D_sh_^*^ curve.

A quasi‐2D perovskite‐organic (BA_2_MA_4_Pb_5_I_16_‐P3HT: PC_71_BM) coupling separation structure was established to fabricate a series of PM‐NPDs, the schematic diagram of the photodetectors structure and the chemical structures of the used materials are shown in Figure 1a and Figure S1. Evidently, cross‐sectional scanning electron microscope (SEM) imaging of quasi‐2D perovskite‐organic PM‐NPDs reveals a well‐defined multi‐layer structure with distinct interlayer interfaces, confirming the structural integrity of quasi‐2D perovskite‐organic hybrid coupling in PM‐NPDs (Figure 1b). The energy level alignment quasi‐2D perovskite‐organic PM‐NPD and absorption spectra of perovskite and organic materials are shown in Figure 1c,d. Specifically, quasi‐2D perovskites were employed as the front optical filter, enabling selective transmission of target‐band photons while suppressing short‐wave background response, and the organic layer served as the photoresponse layer. The normalized absorption spectra of the quasi‐2D perovskite filter layer and external quantum efficiency (EQE) of the organic photoresponse layer are presented to elucidate the spectral‐shaping mechanism of the perovskite filter layer, as shown in Figure 1e. Essentially, the photogenerated excitons in the photoresponse layer can be dissociated into free charge carriers at P3HT: PC_71_BM interfaces. Photogenerated holes are transported along P3HT channels and the perovskite layer, then collected by the ITO electrode. Meanwhile, photogenerated electrons are trapped in PC_71_BM due to discontinuous electron transport channels, which induces band bending at the photoresponse layer/Al electrode interface to enable hole tunneling injection. The EQE of photodetectors without a filter exhibited a dual‐peak response at 350 and 600 nm, with an obvious dip in the spectral range of 450–550 nm. This phenomenon can be well explained by the strong photon harvesting of P3HT films and the Beer‐Lambert law, as shown in Figure S2 [29]. We should also note that the donor‐acceptor (P3HT: PC_71_BM) ratio in the organic layer can affect the PM effect. When more PC_71_BM is incorporated in the active layers, part of PC_71_BM may directly connect with the Al electrode, resulting in the photogenerated electrons collected by the Al electrode. If the photogenerated electrons are collected by the Al electrode, which cannot induce interfacial band bending for hole tunneling injection. Therefore, the organic layer in PM‐NPDs with P3HT: PC_71_BM = 100:1 weight ratio was selected to achieve optimal balance. This optimal ratio provides sufficient PC_71_BM to form electron traps, induces strong band bending, and prevents direct contact between the acceptor and the Al electrode, thereby maximizing hole tunneling injection (Figure S2). Thus, low‐bias‐driven PM and high spectral selectivity were simultaneously achieved by coupling the spectral filtering layer and the photoresponse multiplication layer, as illustrated in Figure 1f. Furthermore, the quasi‐2D filter layer exhibited a strong absorption in 300–500 nm, which is an obvious overlap with the response peak at 350 nm of the photoresponse layer. The response was shifted toward the target 600 nm band due to efficient suppression of short‐wavelength photons after integrating the perovskite filter layer, inducing a significant narrowband response. It is also important to note that spectral selectivity and photon utilization efficiency must be balanced in the design of a perovskite filter for quasi‐2D perovskite‐organic PM‐NPDs. Interestingly, the optimized device with 110 nm thickness exhibits a significant narrowband response at about 600 nm (FWHM = 58 nm), achieving 600% EQE and a specific detectivity (D_sh_^*^, D_sh_^*^ = R/(2eJ_D_)^0.5^) of 1.05 × 10^11^ Jones at a low bias of 3 V (Figure 1g,h). This result raises a fundamental structural question whether the perovskite filter layer in tandem‐like perovskite‐organic devices can also be made relatively thin to accommodate future integrated device structures. To investigate the influence of quasi‐2D perovskite filter thickness on narrowband detection ability, the quasi‐2D perovskite‐organic PM‐NPDs with 70, 110, 240, and 350 nm quasi‐2D perovskite filters were fabricated. It can be seen that the devices with 70‐nm quasi‐2D perovskite filters exhibit a response signal in the range of 300–500 nm. This is because PM‐NPDs with a perovskite thickness of 70 nm cannot fully absorb short‐wavelength photons, resulting in residual response in the 300–500 nm range and failing to achieve a significant narrowband response, as shown in Figure S3. When the thickness of perovskite exceeds 240 nm, a large number of photons are absorbed at 600 nm, which reduces the light flux reaching the active layer and decreases the EQE. Compared to the 110 nm perovskite filter, the 350 nm perovskite filter demonstrated higher photon harvesting capability and steeper absorption edges, the photon transmittance of that is restricted by excessive filtration in the target band, impacting the quasi‐2D perovskite‐organic PM‐NPDs performance, as shown in Figure S4a. Notably, the 110 nm quasi‐2D perovskite‐organic PM‐NPDs achieved a peak EQE over 600%, outweighing the 350 nm quasi‐2D perovskite‐organic PM‐NPDs by approximately threefold. The peak D_sh_^*^ and photocurrent density (*J_L_*) of 350 nm quasi‐2D perovskite‐organic PM‐NPDs were obviously lower than that of 110 nm quasi‐2D perovskite‐organic PM‐NPDs, as shown in Figure 1h and Figure S4b. It should be noted that this result indicates that comparable performance of quasi‐2D perovskite‐organic PM‐NPDs with a thinner perovskite filter layer can be realized via quasi‐2D perovskite‐organic layer coupling.

**FIGURE 2:**
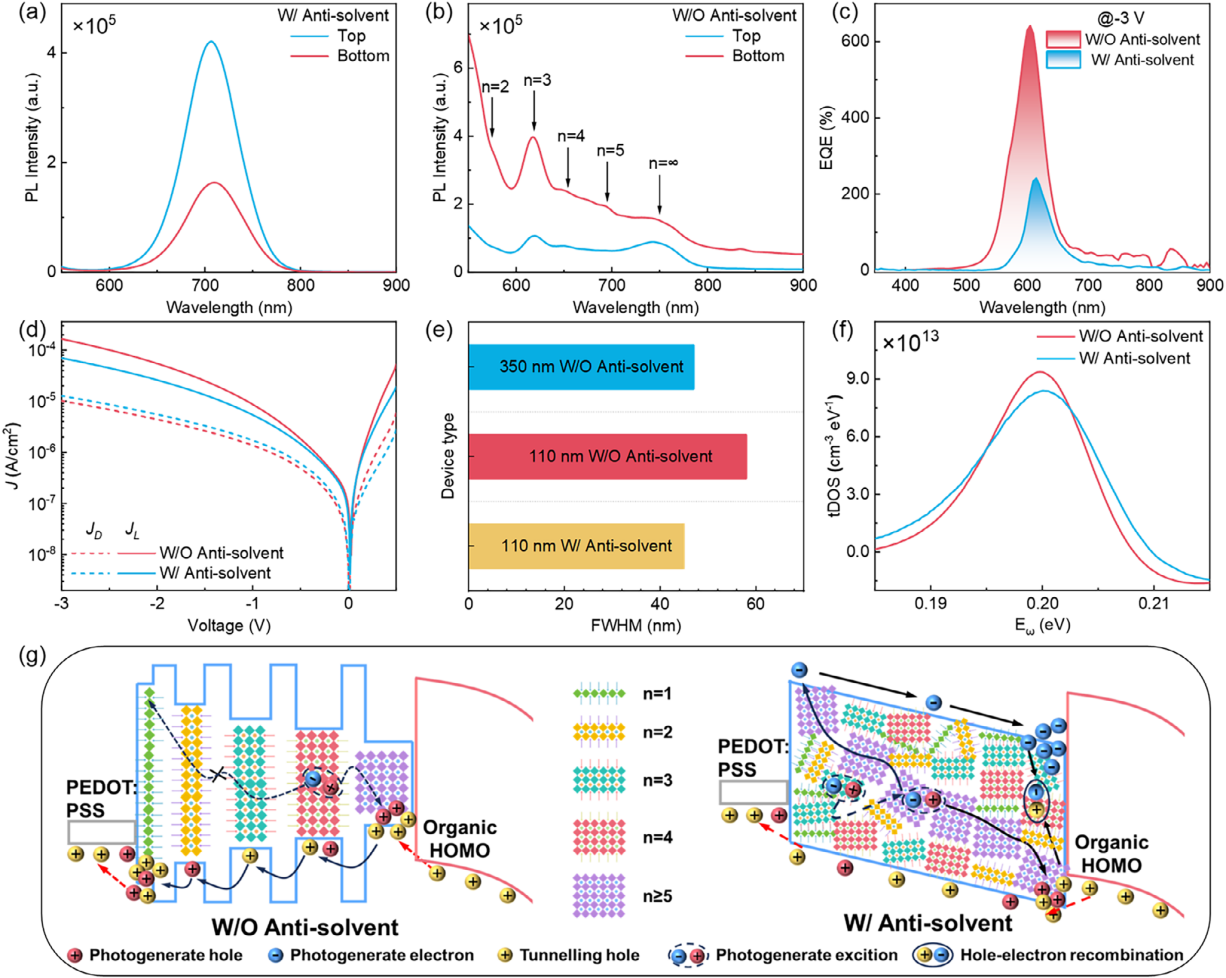
PL spectra of quasi‐2D perovskite films excited from top and bottom surface (a) with anti‐solvent‐assisted; (b) without anti‐solvent‐assisted. Quasi‐2D perovskite‐organic PM‐NPDs prepared with and without anti‐solvent‐assisted (c) EQE spectra measured at −3 V; (d) *J*–*V* curve; (e) FWHM; (f) tDOS. (g) Schematics of charge dynamics of quasi‐2D perovskite‐organic PM‐NPDs prepared without (left) and with (right) anti‐solvent‐assisted.

To gain insights into the distribution of quasi‐2D perovskite multiple quantum wells on the performance of quasi‐2D perovskite‐organic PM‐NPDs, a comparative analysis was conducted on quasi‐2D perovskite (BA_2_MA_4_Pb_5_I_16_) films fabricated with and without anti‐solvent‐assisted crystallization methods. Normally, quasi‐2D perovskite films typically consist of nanoplates with varying n‐values, where n denotes the number of inorganic perovskite sheets in the quantum well formed during the crystallization process [30, 31, 32]. Due to their relatively lower intrinsic formation energy, low‐n phases tend to crystallize initially, whereas high‐n phases, which demand higher formation energy, exhibit slower growth rates. The optical properties of quasi‐2D perovskites are largely determined by energy transfer between these different‐n‐value nanoplates [33]. Specifically, the spatial arrangement of these nanoplates in quasi‐2D films can be categorized into two main configurations: the uniform dispersion of different‐n‐value nanoplates (in anti‐solvent processed films) and the non‐uniform dispersion (in films without anti‐solvent processing). In the case of non‐uniform dispersion, the nanoplates exhibit an ordered distribution along the vertical direction of the film, from smaller n‐values near the bottom surface to larger n‐values toward the top surface [34]. For instance, such films may contain nanoplates with n = 1 to 5 arranged sequentially from bottom to top. This vertical ordering enables cascade energy transfer from n = 1 to 5 nanoplates (from the bottom to the top of the film) but inhibits energy transfer in the reverse direction (from top to bottom). In contrast, uniform dispersion is characterized by a homogeneous mixture of different‐n‐value nanoplates throughout the film. This homogeneous arrangement increases the likelihood of direct contact between small‐n‐value nanoplates and large‐n‐value nanoplates, facilitating isotropic energy transfer from small‐n‐value nanoplates directly to n = 5 nanoplates, which act as the primary light‐emitting centers in the film. To distinguish between these two configurations, PL measurements were performed on the quasi‐2D perovskite films under 500 nm excitation, with the excitation source directed at either the bottom or top surface of the films. As shown in Figure 2a, the films prepared with anti‐solvent processing exhibited similar PL spectra regardless of whether excitation was applied from the bottom or top surface. In contrast, the films fabricated without anti‐solvent processing showed distinct PL spectra when excited from the two different directions (Figure 2b). These results indicate that the different‐n‐value nanoplates are uniformly dispersed in the anti‐solvent‐treated quasi‐2D perovskite films, while they adopt a non‐uniform distribution in the films prepared without anti‐solvent processing. Evidently, anti‐solvent processing accelerates the crystallization of quasi‐2D perovskite films, and this accelerated crystallization in turn promotes the uniform dispersion of nanoplates with varying n‐values within the films. The anti‐solvent treated based quasi‐2D perovskite contains a substantial amount of >5 nanoplates phases within the overall film thickness, verified by the PL results. Importantly, an interesting observation emerges from the quasi‐2D film fabricated without anti‐solvent processing, as it exhibits enhanced PM EQE response characteristics (Figure 2c). It also shows an increase in the *J_L_* and a decrease in dark current density (*J_D_*), with a FWHM of 58 nm in quasi‐2D perovskite‐organic PM‐NPDs fabricated without anti‐solvent processing, as compared to the PM‐NPDs fabricated with anti‐solvent processing, which exhibit a FWHM of 44 nm, as shown in Figure 2d,e. Notably, there is a clear distinction in the trap density of states (tDOS) between uniform and non‐uniform dispersion quasi‐2D perovskite films, where the uniform films exhibit low tDOS and the non‐uniform ones show high tDOS, respectively (Figure 2f). This difference in tDOS indicates defect density variations, where uniform dispersion of different‐n‐value nanoplates correlates with lower defect density and non‐uniform dispersion with higher defect density. Thus, this finding suggests that PM in quasi‐2D perovskite‐organic PM‐NPDs depends on whether anti‐solvent processing is employed to form a favorable nanoplate dispersion in the perovskite filter layer. We should note that the film thickness without and with anti‐solvent treatment is 129 and 121 nm, respectively. This indicates that the performance difference between the quasi‐2D perovskite‐organic PM‐NPDs without and with anti‐solvent treatment arises from the dispersion state of the nanoplates. In Figure 2g, the schematics of charge dynamics of quasi‐2D perovskite‐organic PM‐NPDs prepared without and with anti‐solvent‐assisted are presented to clarify that the structural characteristics of quasi‐2D perovskite and their interfacial behaviors with organic layers are mainly responsible for the decrease in multiplication EQE of quasi‐2D perovskite/organic detectors. In a non‐uniform dispersion quasi‐2D perovskite nanoplate structure, the contact interface between perovskite and organic layer is dominated by the interaction between narrow‐bandgap perovskites and organic materials, and this cascade transport creates a significant barrier to charge transport toward the perovskite/organic interface. Specifically, the conduction band offset, which refers to the difference between the lowest unoccupied molecular orbital (LUMO) of narrow‐bandgap perovskite and that of the organic layer, is considerably larger. This larger LUMO energy barrier inhibits the migration of trapped electrons to the perovskite/organic interface, thereby suppressing the interfacial charge recombination process detrimental to the PM effect and further boosting the EQE multiplication. In contrast, for uniform dispersion quasi‐2D perovskite nanoplate structures, the perovskite/organic contact interface features the coexistence of both wide‐bandgap and narrow‐bandgap perovskites in contact with organic layers. The energy of wide‐bandgap perovskites can be efficiently and rapidly transferred to narrow‐bandgap perovskites, which promotes the transport of charges to the perovskite/organic interface and creates favorable conditions for interfacial charge recombination. Moreover, it is widely recognized that non‐radiative recombination and inadequate energetic alignment at the wide‐bandgap (approximately 1.8 eV, bromide‐rich perovskite) perovskite/C60 interface lead to halide phase segregation under continuous irradiation within the devices [35]. In addition, the direct contact between wide‐bandgap perovskites and organic layers also leads to a closer match between their LUMO and highest occupied molecular orbital (HOMO) energy levels. This alignment forms a continuous charge transport channel that facilitates the direct transport of charges through the interface rather than suppressing the charge multiplication process via interfacial recombination, ultimately weakening the PM effect and reducing its multiplication EQE in quasi‐2D perovskite‐organic PM‐NPDs. As a result, quasi‐2D perovskite with non‐uniform dispersion plays two key roles in enabling PM in quasi‐2D perovskite‐organic detectors by modulating the response band with a relatively thin absorber and accelerating the charge transport process. In contrast, quasi‐2D perovskites with uniform dispersion fail to sustain effective PM in quasi‐2D perovskite‐organic detectors because their disordered structure disrupts both the response band regulation and hole transport process required for the PM phenomenon.

**FIGURE 3:**
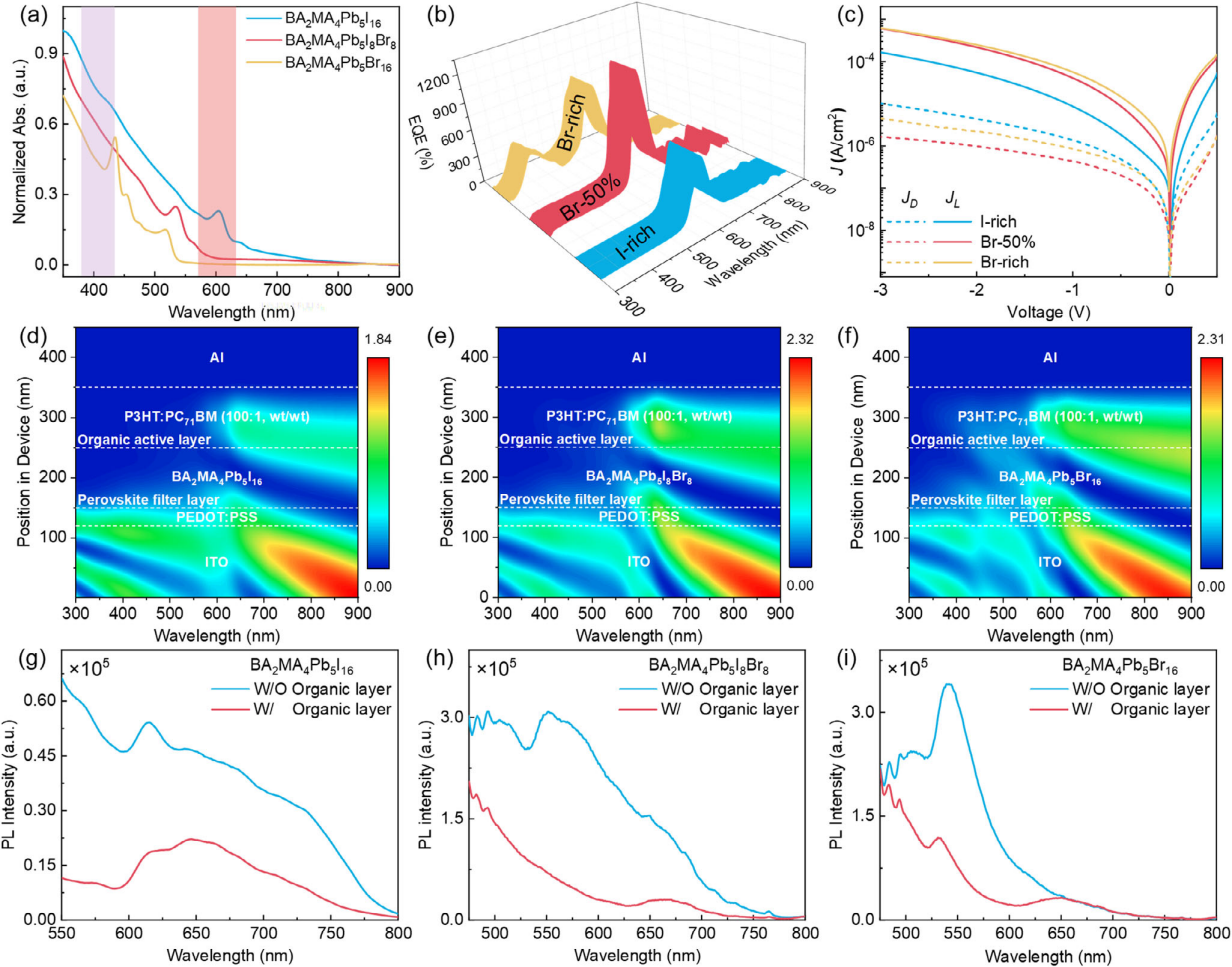
(a) The normalized absorption spectra of quasi‐2D perovskite films based on BA_2_MA_4_Pb_5_I_16_ (I‐rich), BA_2_MA_4_Pb_5_I_8_Br_8_ (Br‐50%), and BA_2_MA_4_Pb_5_Br_16_ (Br‐rich). Quasi‐2D perovskite‐organic PM‐NPDs based on I‐rich, Br‐50%, and Br‐rich (b) EQE spectra; (c) *J*–*V* curve. Optical field profiles of PM‐NPDs based on (d) I‐rich; (e) Br‐50%; and (f) Br‐rich. PL spectra of quasi‐2D perovskite films with and without organic PM‐layer based on (g) I‐rich; (h) Br‐50%; and (i) Br‐rich.

To explore the regulatory effect of halide composition on quasi‐2D perovskite‐organic PM‐NPDs performance, the filter characteristics were optimized by tuning the halogen ratio. The filter layers based BA_2_MA_4_Pb_5_I_16_ (I‐rich), BA_2_MA_4_Pb_5_I_8_Br_8_ (Br‐50%), and BA_2_MA_4_Pb_5_Br_16_ (Br‐rich) were prepared, and their absorption spectra are presented in Figure 3a. A gradual blue shift in the absorption edge was observed with increasing bromide (Br) content, leading to a significant reduction in absorption within 300–500 nm range. This allowed a portion of short‐wavelength light to penetrate the filter layer and reach the photoresponse layer, thereby generating a photoresponse. The EQE spectra of quasi‐2D perovskite‐organic PM‐NPDs with varying Br content were measured and are shown in Figure 3b. The I‐rich quasi‐2D perovskite‐organic PM‐NPDs exhibited a typical narrowband response with a peak EQE of 600% and a FWHM of 58 at 600 nm. Upon increasing the bromine content to 50%, the peak EQE of the Br‐50% quasi‐2D perovskite‐organic PM‐NPDs increased to 1200%, with the FWHM slightly broadened to 60 nm, while maintaining a well‐defined narrowband response centered at 600 nm. The higher EQE observed in Br‐50% perovskite‐based devices, as compared to their I‐rich counterparts, can be primarily attributed to a more favorable alignment between the bandgap and the incident photon energy. This improved spectral match enhances light absorption efficiency, effectively compensating for the relatively lower carrier collection efficiency typically associated with I‐rich materials. The enhanced carrier generation in the target band is likely due to the appropriate spectral filtering effect provided by the Br‐50% configuration. Furthermore, the Br‐rich perovskite‐based photodetectors exhibit a distinct dual‐peak photoresponse at 380 and 600 nm. This behavior is well explained by the increased penetration of short‐wavelength photons into the photoresponse layer, leading to efficient generation of photocarriers in the UV region. Moreover, *J*–*V* characteristics of PM‐OPDs with different filter layers were systematically measured in dark and under 0.7 mW/cm^2^ white light illumination conditions, as shown in Figure 3c. The *J_L_* of quasi‐2D perovskite‐organic PM‐NPDs exhibits a monotonically increased trend along with increasing Br content, which is attributed to occur more photogenerated exciton dissociation. The *J_D_* of quasi‐2D perovskite‐organic PM‐NPDs exhibits a trend wherein the I‐rich composition demonstrates higher values compared to Br‐rich and Br‐50% compositions, which is likely attributable to variations in charge transport properties arising from differences in the filter layers.

To systematically elucidate the filtering characteristics of perovskite filter layers with varying bromine compositions, the optical field distributions in quasi‐2D perovskite‐organic PM‐NPDs were simulated using the transfer matrix method, as illustrated in Figure 3d–f [36]. The refractive indices (n) and extinction coefficient (k) were determined using an ellipsometry polarimeter, as shown in Figure S5. Due to the blue‐shift effect induced by Br doping, the transmission of short‐wavelength light was enhanced, thereby increasing the photon flux reaching the photoresponse layer, as evidenced by the transmitted spectrum observed (Figure S6). Meanwhile, distinct optical interference fringes are observed in the weak absorption region of quasi‐2D perovskite‐organic PM‐NPDs, resulting from the interaction between incident light and light reflected from the Al electrode. The simulated optical field intensity distributions for 400, 500, and 600 nm illumination are presented in Figure S7. With increasing bromine content, the optical intensity is enhanced across the entire device structure, particularly within the photoresponse layer, which effectively accounts for the secondary photoresponse peak observed at 380 nm in Br‐rich quasi‐2D perovskite‐organic PM‐NPDs. Based on the simulated optical field profiles, the spatial distribution of trapped electrons was further calculated, as illustrated in Figure S8a–c. The trapped electron density in the photoresponse layer increases significantly with higher bromine incorporation, attributed to enhanced photon absorption and subsequent generation of additional electrons captured by PC_71_BM. According to the organic PM mechanism, a higher concentration of trapped electrons near the Al electrode strengthens the Coulombic field at the Al/organic interface, thereby inducing greater interfacial band bending and promoting hole tunneling injection, which enhances device responsivity. The wavelength‐dependent trapped electron density profiles, extracted from cross‐sectional regions located 10 nm adjacent to the Al electrode, are shown in Figure S8d. It is evident that the trapped electron density curve for Br‐50% exhibits higher values compared to those of Br‐rich and I‐rich compositions, which is consistent with the EQE in both magnitude and spectral shape. Moreover, the tDOS of PM‐NPDs based on I‐rich, Br‐50%, and Br‐rich were measured to further confirm the variation in defect density with halogen composition. (Figure S9). This observation is in good agreement with the organic PM mechanism and emphasizes the significance of adjusting the bromine content for optimizing the device response. Moreover, it can be observed that a blue shift of the PL spectra occurs in quasi‐2D perovskite films from I‐rich to Br‐50% and Br‐rich compositions, as well as a significant decrease in PL intensity in I‐rich, Br‐50%, and Br‐rich quasi‐2D perovskite films compared to those without an organic photoresponse layer. These observations further confirm that charge transfer and suppressed radiative recombination contribute to enhanced EQE in devices where the emission spectrum is regulated by halogen ratio (Figure 3g–i).

**FIGURE 4:**
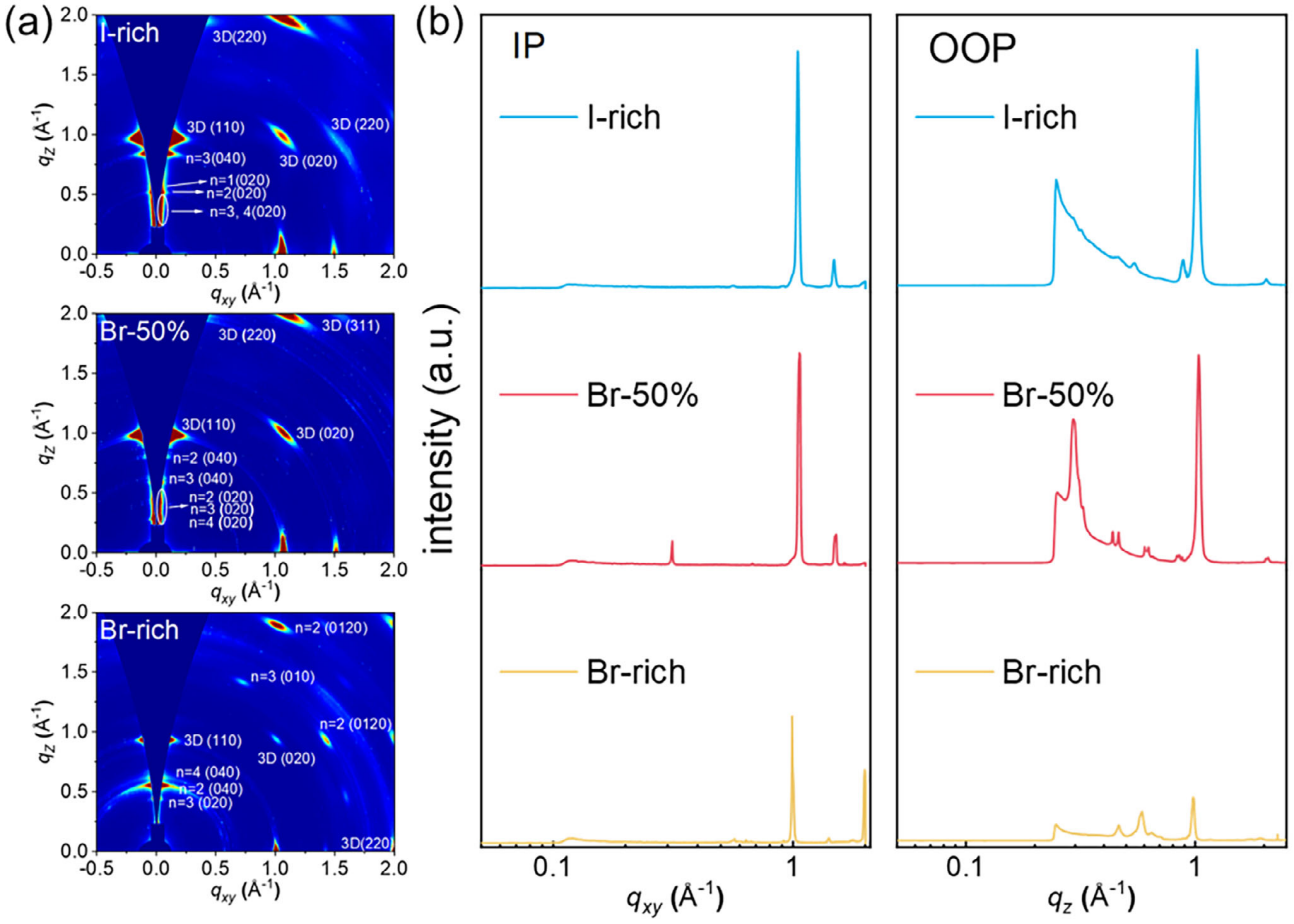
Quasi‐2D perovskite films with different halide composition ratios of I‐rich, Br‐50%, and Br‐rich (a) The 2D‐GIWAXS patterns; (b) 1D line‐cuts profiles of the out‐of‐plane and in‐plane extracted from the corresponding 2D‐GIWAXS patterns.

To gain a deeper understanding of how halide composition influences molecular‐level morphology, grazing‐incidence wide‐angle X‐ray scattering (GIWAXS) measurements were conducted to investigate molecular crystallinity and packing behavior in quasi‐2D perovskite‐organic PM‐NPDs with varying halide composition ratios. The corresponding 2D GIWAXS patterns and 1D line‐cut profiles of quasi‐2D perovskite with I‐rich, Br‐50%, and Br‐rich compositions are presented in Figure 4. The I‐rich film exhibits intense and well‐defined diffraction peaks, indicating high crystallinity and a pronounced preferred orientation along both the in‐plane (IP) and out‐of‐plane (OOP) directions. The Br‐50% film exhibits moderate peak intensity and a relatively uniform distribution, indicating a reduction in crystallographic order relative to the I‐rich film. In contrast, the Br‐rich sample displays weak and broad diffraction peaks, indicative of significantly diminished crystallinity and preferred orientation. In Figure 4b, 1D line‐cut profiles quantify crystallinity and orientation, revealing a peak intensity sequence of I‐rich, Br‐50, and Br‐rich that confirms I‐rich exhibits the highest crystallinity. Notably, the I‐rich films prominent IP peak signals a favorable in‐plane orientation, beneficial for in‐plane carrier transport, which is critical for detector photosensitive layers. According to the Scherrer formula, the CCLs in 100 (IP) and 010 (OOP) of the I‐rich film, Br‐50%, and Br‐rich are 188 and 118 Å, 171 and 136 Å, 353 and 184 Å, respectively (Table S2). The I/Br compositional variation modulates lattice parameters due to I^−^ ionic radius larger than Br. I‐rich corresponds to a larger lattice spacing, while Br‐rich corresponds to a smaller lattice spacing. In quasi‐2D perovskite‐organic PM‐NPDs, I‐rich show high crystallinity and preferred in‐plane orientation, providing a robust structural basis for efficient carrier transport and reduced defect‐mediated recombination. In contrast, increasing Br content degrades crystallographic order, necessitating a trade‐off between band‐gap tuning and structural integrity.

**FIGURE 5:**
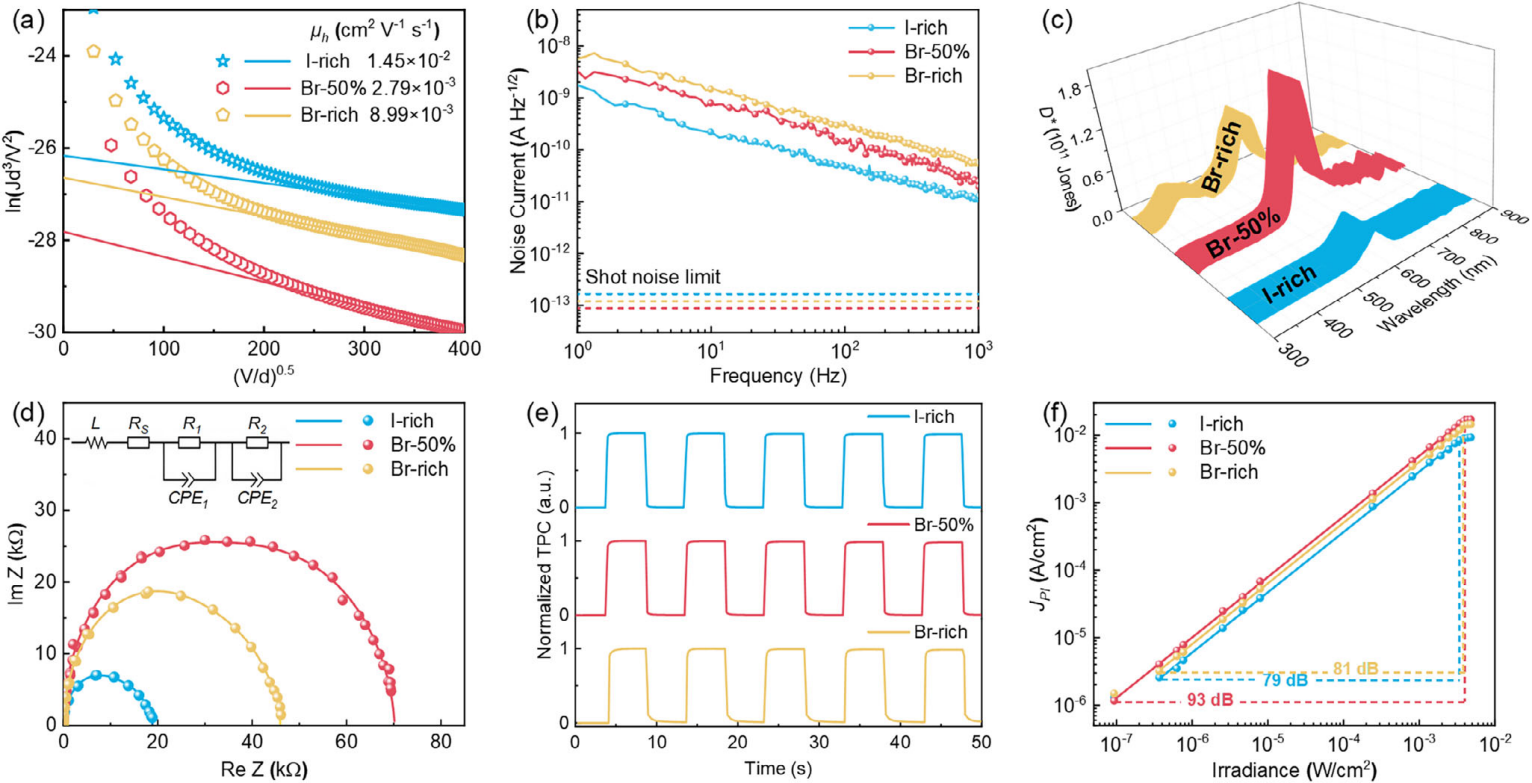
The quasi‐2D perovskite‐organic PM‐NPDs based on I‐rich, Br‐50%, and Br‐rich measured at −3 V; (a) Hole mobility from SCLC method; (b) Noise current spectra; (c) D^*^ spectra; (d) Nyquist plots; (e) Normalized TPC curves; (f) LDR.

To further investigate the structural effect of halide composition on the charge‐transport properties of the quasi‐2D perovskite‐organic PM‐NPDs, a series of charge transport characterization were carried out. The hole mobility was evaluated by analyzing the ln (Jd^3^/V^2^) versus (V/d)^0.5^ characteristics based on a structure of ITO/PEDOT: PSS/Perovskite/Organic BHJ/MoO_3_/Al, as shown in Figure 5a. The highest hole mobility was observed in the I‐rich devices (1.45 × 10^−2^ cm^2^/V s), while the lowest was recorded for the Br‐50% devices (2.79 × 10^−3^ cm^2^/V s). This trend correlates well with crystallinity and crystal orientation as enhanced crystallinity in the I‐rich film reduces carrier scattering, thereby facilitating more efficient charge transport. Moreover, the noise current (*i_n_*) of various quasi‐2D perovskite‐organic PM‐NPDs was measured at 3 V, as shown in Figure 5b. The I‐rich device exhibits the lowest noise current, attributable to its low defect density resulting from high crystallinity, whereas the Br‐rich device shows the highest noise level due to the presence of abundant defects. Furthermore, the D^*^ spectra of are obtained and shown in Figure 5c. D^*^ is a critical parameter for photodetectors, commonly used to evaluate their detection performance. Based on the *i_n_*, D^*^ can be calculated using the following equations [37, 38]:

$$\;D^{*}=\frac{\sqrt{A}}{NEP}\;\left(cm\;Hz^{0.5}\;W^{-1}\;or\;Jones\right)$$

$$NEP=\frac{i_{n}}{R\sqrt{B}}\;\left(W\;Hz^{-0.5}\right)$$

where A denotes the photoactive area, B represents the electrical bandwidth, NEP refers to the noise equivalent power, and R signifies the responsivity, as exhibited in Figure S10. It is evident that the quasi‐2D perovskite‐organic PM‐NPDs based on Br‐50% exhibit the highest D^*^ value, which can be attributed to their lower *i_n_* and higher R. Figure 5d presents the electrochemical impedance spectroscopy (EIS) measured at 3 V, revealing that the I‐rich and Br‐50% based devices exhibits the lowest and highest charge transport resistance, which can be attributed to its well‐ordered crystalline structure. Meanwhile, within two distinct frequency ranges, these can be modeled and interpreted as two separate R‐C circuits connected in series (inset of Figure 5d). The lower frequency part refers to the chemical capacitance (C_µ_) and recombination resistance (R_recomb_) for the excess electron‐hole carriers, while the higher frequency part is associated with the hopping/drift mechanism for the carriers [39, 40]. It can be seen that Br‐50% devices demonstrate the lowest C_µ_ and highest R_recomb_ than I‐rich and Br‐rich, which is consistent with the above performance of Br‐50% devices (Table S3). Specifically, the transient photocurrent (TPC) of various quasi‐2D perovskite‐organic PM‐NPDs was measured under 590 nm illumination at a bias voltage of 3 V, as shown in Figure 5e. The rise time (*t_r_*) and fall time (*t_f_*) were defined as the time required for the transient photocurrent to increase from 10% to 90%, and to decrease from 90% to 10%, of its maximum value, respectively [41, 42, 43]. As illustrated in Figure S11, the TPC of I‐rich demonstrates more efficient carrier transport and reduced recombination, consistent with its high crystallinity. In contrast, Br‐rich displays a slower TPC response, which is relatively indicative of lower hole mobility. It should be noted that the linear dynamic range (LDR) is a critical parameter for photodetectors, as it quantifies their ability to detect light intensities across a wide range of magnitudes. The LDR of a photodetector can be determined using the following formula: [44, 45, 46, 47]

$$LDR=20log\frac{I_{upper}}{I_{lower}}\;\left(dB\right)$$

where *I_upper_* and *I_lower_* represent the maximum and minimum detectable light intensity within the linear dynamic range, respectively. The *J_PI_* values of quasi‐2D perovskite‐organic PM‐NPDs with I‐rich, Br‐50%, and Br‐rich compositions were measured under 590 nm illumination at different light intensities and a fixed bias of 3 V, as illustrated in Figure 5f. Br‐50% devices exhibit superior linearity with respect to irradiance, which can be attributed to its lower *J_D_* and favorable crystalline structure. Specifically, the I‐rich device achieves the highest hole mobility and the lowest noise current due to its high crystallinity, yet its low trap density leads to insufficient electron trapping efficiency, weak interfacial band bending, and thus limited PM effect from hole tunneling injection. In contrast, the Br‐50% device, with moderate crystallinity reduction and increased trap states induced by Br doping, not only optimizes energy level alignment via the I/Br halogen gradient to boost photon absorption and carrier generation in the target band, but also forms a higher trapped electron density through moderate defects to strengthen interfacial band bending, thereby greatly promoting the PM effect and realizing a significant EQE improvement. Moreover, the reproducibility of peak EQE, *J_D_*, R, for quasi‐2D perovskite‐organic PM‐NPDs based on I‐rich, Br‐50%, and Br‐rich was evaluated to further confirm the reliability of the results (Figure S12). Overall, these results are consistent with the structural insights obtained from GIWAXS analysis, which demonstrates the effect of halide compositional modulation in the quasi‐2D perovskite filter layer on both the material structure and device performance.

## 3 Conclusion

In summary, low‐power, high‐selectivity PM‐NPDs is achieved by establishing a quasi‐2D perovskite‐organic coupled separated structure. Essentially, the quasi‐2D perovskite layer serves as an efficient charge transport regulator and intrinsic optical filter, while the organic BHJ functions as the photoresponse layer for the PM effect. Specifically, anti‐solvent‐free preparation yields a quasi‐2D perovskite layer with non‐uniform dispersion of different‐n‐value nanoplates, which inhibits directional energy transfer and interfacial recombination of tunneling‐injected holes to enhance the PM effect. In contrast, a quasi‐2D perovskite with uniform dispersion prepared using anti‐solvent processing reduces the defect density but weakens the PM effect. Moreover, thickness optimization shows that a 110 nm quasi‐2D perovskite layer achieves the best balance between spectral selectivity and photon utilization efficiency, enabling a thinner device without external filters while maintaining an EQE over 600%. Furthermore, halide composition engineering via I/Br substitution precisely tailors the perovskite bandgap and absorption edge, enabling selective transmission of 600 nm target photons, suppressing 300–500 nm short‐wavelength background response, and enhancing spectral matching with incident photons through Br‐induced absorption edge blue shift. However, excessive Br ratio degrades crystallinity and deteriorates carrier transport properties. Consequently, the 50% Br device achieves the optimal balance with a peak EQE of 1200%, FWHM of 60 nm, D^*^ of 1.81 × 10^11^ Jones, and low noise current, revealing the critical trade‐off between bandgap alignment and crystallinity in halide composition tuning. This study not only demonstrates the effective integration of perovskite‐based filtering and organic PM but also presents innovative device architectural strategies for advancing low‐power, high‐spectral‐selectivity, and high‐gain photodetection systems.

## 4 Methods

### 4.1 Device Fabrication

The devices were developed with a structure of the ITO/PEDOT: PSS/Perovskite/ active layer/Al. The preparation steps were carried out in the following sequence: The ITO‐coated glass substrates (resistance 7 ohms per square) were cleaned in an ultrasonic bath sequentially with a cleaner, deionized water, isopropyl alcohol, and ethanol. The cleaned glass substrates were dried with nitrogen and stored in an oven at 50 °C. Before device fabrication, the substrates were treated with UV ozone plasma for 180s. Weigh 32.17 mg of BAI, 50.87 mg of MAI, and 184.40 mg of PbI_2_ (or 24.65 mg of BABr, 35.83 mg of MABr, 146.80 mg of PbBr_2_; or 32.17 mg of BAI, 50.87 mg of MAI, 36.88 mg of PbI_2_, and 117.44 mg of PbI_2_), and dissolve them in 1 mL of a mixed solvent of DMSO and DMF (DMSO: DMF = 1:19). Stir the solution at 25 °C for 4 h. Prepare a PC_71_BM and P3HT solution with a concentration of 10 and 30 mg/ml in chloroform at a ratio of 1:100. Filtered PEDOT: PSS is uniformly coated on a clean ITO glass substrate, spin‐coated at 5000 rpm for 45 s, and annealed at 150 °C for 15 min to form an ultra‐thin film. The PEDOT: PSS‐coated substrate is transferred to a nitrogen glove box to prepare an active layer. The dissolved perovskite solution is spread on the PEDOT: PSS layer and spin‐coated at 3000 rpm for 40s to form a perovskite film about 100 nm thick and directly annealed at 100 °C for 10 min. The organic layer was spin‐coated on the perovskite layer at 2000 rpm and annealed at 100 °C for 10 min. Finally, Al (100 nm) was deposited through thermal deposition as the contact metal electrode. The active area of the devices was ≈0.0365 cm^2^.

### 4.2 Device Characterization

The current density versus voltage (*J*–*V*) curves of PM‐NPDs were measured by using a Keithley 2400 source meter. The used monochromatic light was provided by a 150 W xenon lamp coupled with a monochromator. The light intensity spectrum of monochromatic light was recorded by using a Thorlabs S120VC power meter. The UV–Vis absorption spectra of perovskite, P3HT, and PC_71_BM were recorded by a SHIMADZU UV‐3101 PC spectrophotometer. The transient photocurrent of PM‐NPDs based on various perovskite filters was carried out on the Zahner Electrochemical Workstation. The refractive index (n) and extinction coefficient (k) of the blend film were measured by a J.A. Woollam RC2‐XI spectroscopic ellipsometer. Photoluminescence (PL) spectra of films were measured by a HORIBA Fluorolog‐3 spectrofluorometer system.

## Conflicts of Interest

The authors declare no conflicts of interest.

## Supporting information




**Supporting File**: advs75123‐sup‐0001‐SuppMat.docx.

## Data Availability

Data available within the article and supplementary materials.
