# Supplementary material for: Ultrathin Quasi‐2D Perovskite‐Organic Segregated‐Structure Coupling for High‐Performance Narrowband Photomultiplication Detectors
Source: Adv Sci (Weinh). 2026 Apr 2;13(36):e75123. doi: 10.1002/advs.75123 (PMC13317595; doi:10.1002/advs.75123)
Supplement: Supplementary file 1 — Supporting File: advs75123‐sup‐0001‐SuppMat.docx. [file ADVS-13-e75123-s001.docx]

# Supporting Information

# Ultrathin Quasi-2D Perovskite-Organic Segregated-Structure Coupling for High-Performance Narrowband Photomultiplication Detectors

Hongfei Qu^1,2^, Peng He^1,2^, Xingchao Zhao^1,2^, Yongchao Xie^1,2^, Fulong Huang^1,2^, Xiaoling Ma^1,2^, Byung Hui Lee^3^, Han Young Woo^3^, Yumeng Shi^1,2^, Zuliang Zhuo^1,2*^, Fujun Zhang^1,2*^ and Xixiang Zhu^1,2*^

*^1^ Key Laboratory of Luminescence and Optical Information, Ministry of Education, School of Physical Science and Engineering, Beijing Jiaotong University, Beijing 100044, China*

*^2^ Tangshan Research Institute of Beijing Jiaotong University, Tangshan 063000, China*

*^3^ Organic Optoelectronic Materials Laboratory, Department of Chemistry, College of Science, Korea University, 02841, Seoul, Republic of Korea.*

*Corresponding authors:

Zuliang Zhuo:

Email: zhuozuliang@sri-i.com

Fujun Zhang:

Email: [fjzhang@bjtu.edu.cn](mailto:xxzhu@bjtu.edu.cn)

Xixiang Zhu:

Email: [xxzhu@bjtu.edu.cn](mailto:xxzhu@bjtu.edu.cn)

^†^ H. Qu and P. H contribute equally to this work.

**1. Tables**

**Table S1.** Key parameters of typical PM-NPDs

| **Device structure** | **Peak (nm)** | **FWHM**  **(nm)** | **EQE (%)** | **R**  **(A/W)** | **D***  **(Jones)** | **Bias (V)** | **Ref.** |
| --- | --- | --- | --- | --- | --- | --- | --- |
| ITO/PEDOT:PSS/Al₂O₃/P3HT:PC₇₀BM/Al | 650 | 40 | 2.51×10⁵ | 1334 | 9.73×10¹³ | 60 | [1] |
| Ag/MeO-TPD/HAT(CN)₆/D6:C₆₀  /n-Hatna-Cl₆/Ag | 1092 | 18 | 3000 | 15 | 3×10¹¹ | -9 | [2] |
| TiO₂/FTO/cm-TiO₂/mp-TiO₂/BA-BDP/Spiro-OMeTAD/MoO₃/Ag | 510 | 45 | 534 | 157 | 1.9×10¹³ | -3 | [3] |
| ITO/ZnO/PM6/P3HT:PC₇₀BM/Ag | 680 | 54 | 5840 | 32 | 1.23×10¹² | -5 | [4] |
| ITO/PFN-Br/P3HT:PC₇₀BM/  P3HT/P3HT:PTB7-Th:PC₇₀BM/Al | 654 | 70 | 2454 | 12.94 | 9.85×10¹² | -45 | [5] |
| Ag/LiF/Ag/ITO/PEDOT:PSS/P3HT:PC₇₁BM/Al | 350 | 33 | 9300 | - | 8.3×10¹¹ | -15 | [6] |
| P-TPD:F8BT/Glass/ITO/TPAFS-7TMA  /P3HT:PC₇₁BM/Ag | 610 | 44 | 3350 | - | 1.2×10¹¹ | -8 | [7] |
| ITO/2PACz/P3HT:PTB7-Th  /P3HT:PY-DT/Al | 790 | 76 | 1350 | 8.6 | 2×10¹² | -10 | [8] |

**Table S2.** Structural parameters obtained by fitting the IP and OOP profiles of various perovskites.

| **Perovskite** |  | **q (Å^-1^)** | **Peak intensity** | **CCL (Å)** |
| --- | --- | --- | --- | --- |
| **I-rich** | In plane (100) | 1.05 | 6066 | 188 |
|  | Out of plane (010) | 1.02 | 17454 | 118 |
| **Br-50%** | In plane (100) | 1.06 | 5513 | 171 |
|  | Out of plane (010) | 1.03 | 15357 | 136 |
| **Br-rich** | In plane (100) | 0.99 | 3259 | 353 |
|  | Out of plane (010) | 0.98 | 3196 | 184 |

**Table S3.** Electrochemical impedance spectroscopy fitting circuit component parameters.

| **Perovskite** | **L** | **RS** | **R1** | **R2** | **CPE1** | **CPE2** |
| --- | --- | --- | --- | --- | --- | --- |
| **I-rich** | 2.09 μH | 12.9 Ω | 10.5 KΩ | 8.13 KΩ | 705 pF | 11.9 nF |
| **Br-50%** | 2.27 μH | 1.51 Ω | 29.3 KΩ | 40 KΩ | 726 pF | 5.29 nF |
| **Br-rich** | 1.78 μH | 7.8 Ω | 30.8 KΩ | 15.5 KΩ | 683 pF | 13.4 nF |

**2. Figures**


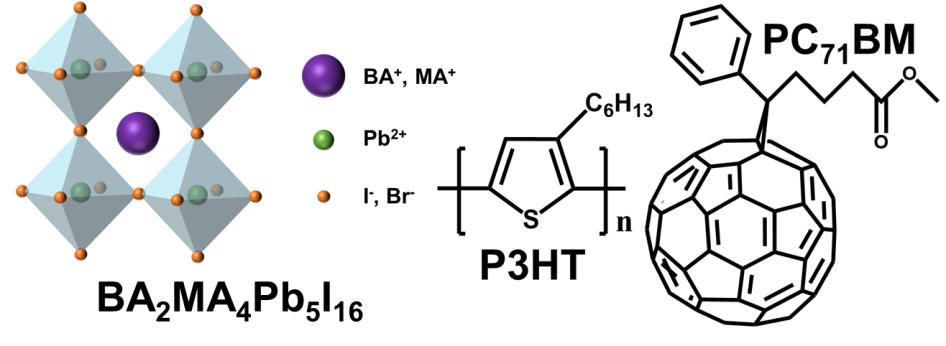


**Figure S1.** The chemical structures of used perovskite and organic materials.


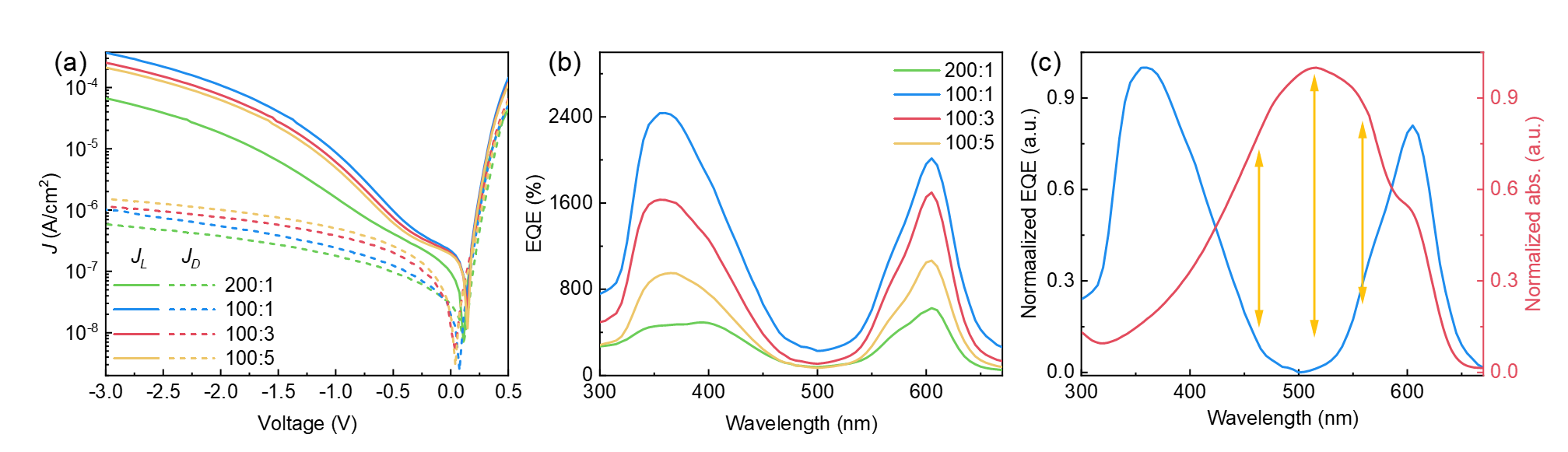


**Figure S2.** (a) The *J-V* curve of the PM-OPDs with different ratio; (b) The EQE spectrum of the PM-OPDs with different ratio; (c) The normalized EQE of PM-OPDs based P3HT:PC71BM (100:1 ,wt:wt) and the normalized absorption spectra of P3HT.

**Figure S3.** The EQE spectrum of PM-NPDs based different perovskite films thickness


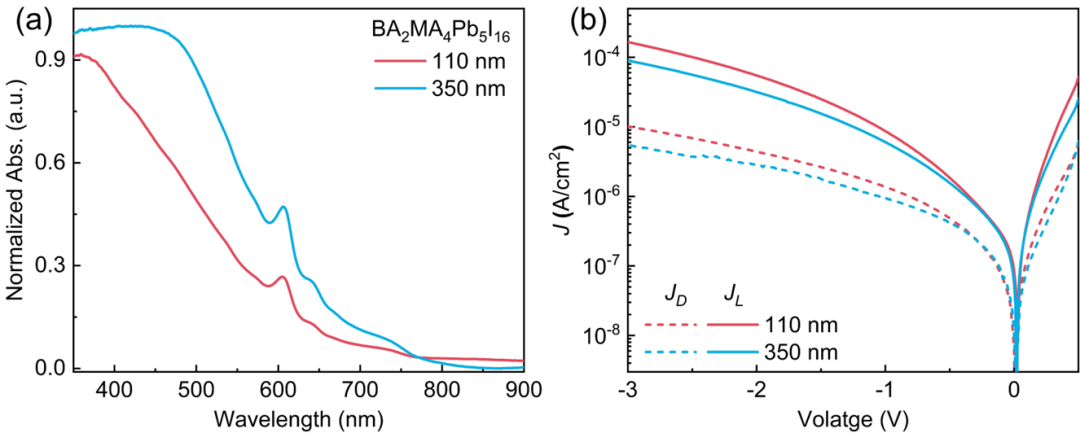


**Figure S4.** (a) The absorption spectrum of different BA_2_MA_4_Pb_6_I_16_ thickness; (b) The *J-V* curve of PM-NPDs based different BA_2_MA_4_Pb_6_I_16_ thickness.


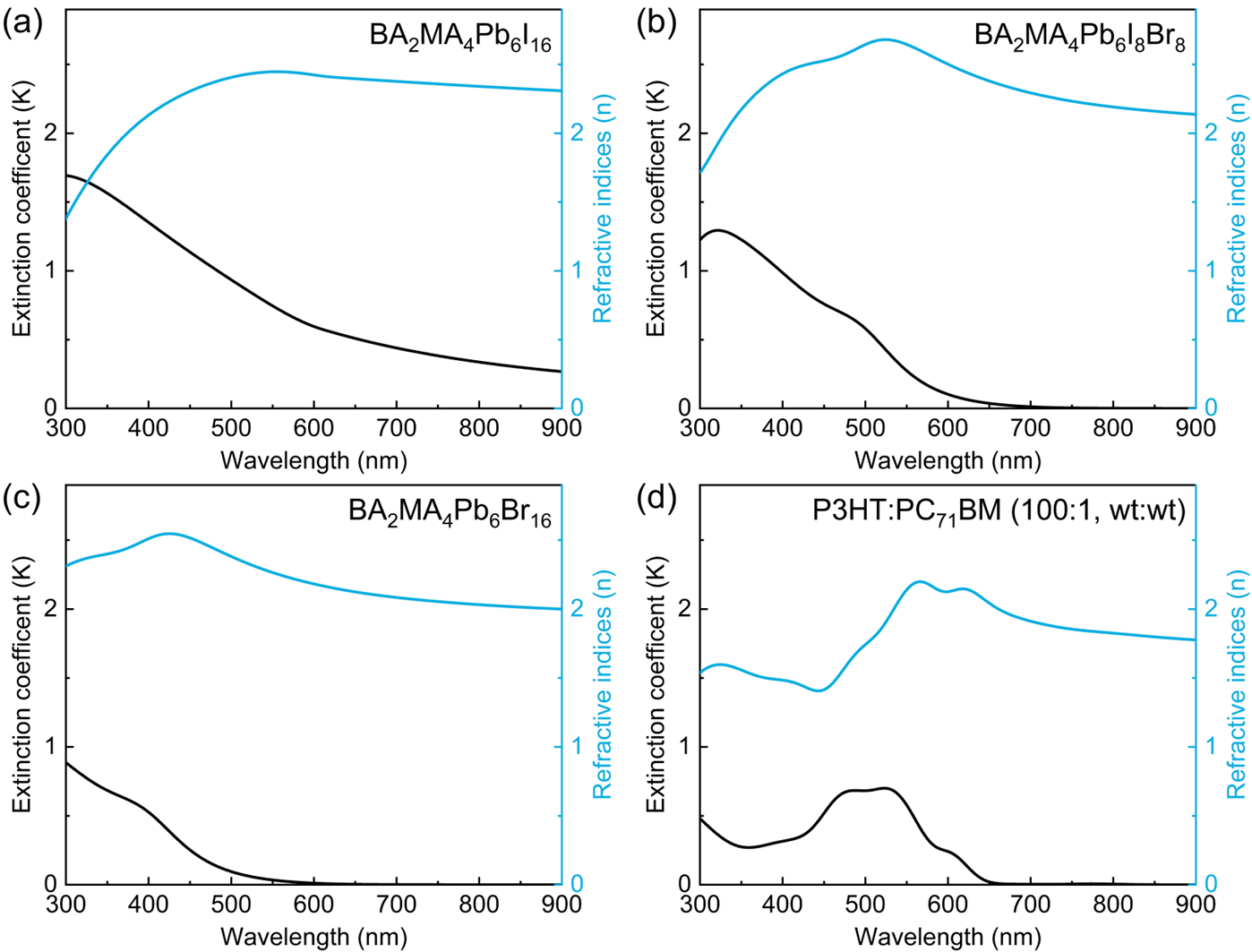


**Figure S5.** *n* and *k* of (a) BA_2_MA_4_Pb_6_I_16_; (b) BA_2_MA_4_Pb_6_I_8_Br_8_; (c) BA_2_MA_4_Pb_6_Br_16_; (d) P3HT:PC71BM (100:1, wt:wt).

The *n* and *k* were provided in **Figure S4**. The field distribution in PM-NPDs was simulated on the basis of *n* and *k* via transfer matrix method (TMM). The trapped electron distribution was calculated according to optical field distribution. Related calculation process is as following.

Each complex index of refraction *n* used in TMM is expressed as Equation (S1):

$\vec{n}=n+ik$ (S1)

Trapped electron density in active layers can be estimated according to Equation (S2) and (S3):

$\rho\left( \lambda\right)\alpha\frac{\left| E\left( \lambda\right) \right|^{2}}{hv}\times\alpha\left( \lambda\right)\times\eta_{D}$(S2)

$$\alpha\left( \lambda\right)=\frac{4\pi k}{\lambda} (S3)$$

where *ρ*(*λ*) is trapped electron density, |*E*(*λ*)|^2^ is optical field intensity, *α*(λ) is absorption coefficient of active layers, and *η_D_* is exciton dissociation coefficient in active layers.


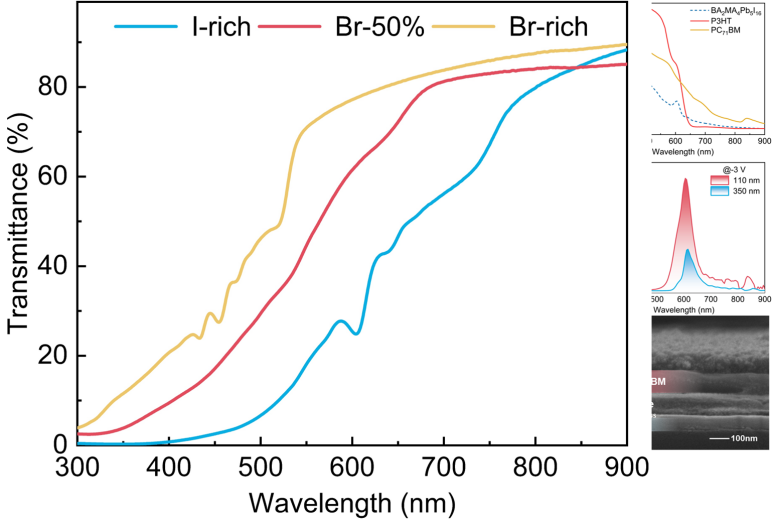


**Figure S6.** The transmission spectrum of various perovskite filter layers.


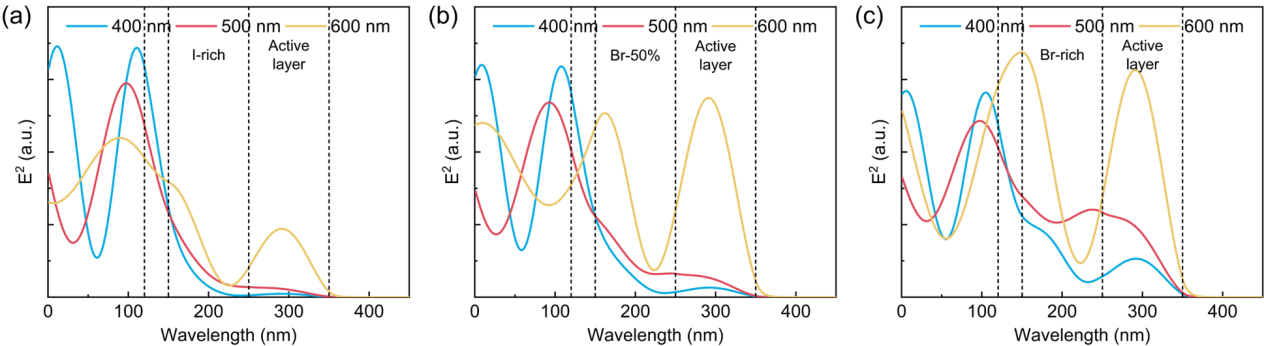


**Figure S7.** The simulated optical field intensity distributions based: (a) BA_2_MA_4_Pb_6_I_16_; (b) BA_2_MA_4_Pb_6_I_8_Br_8_; (c) BA_2_MA_4_Pb_6_Br_16_.


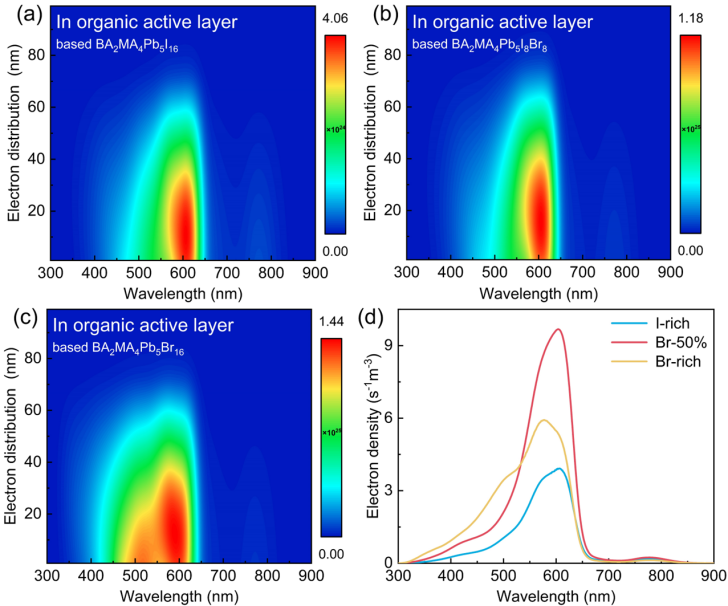


**Figure S8.** The simulated electron density distribution based: (a) BA_2_MA_4_Pb_6_I_16_; (b) BA_2_MA_4_Pb_6_I_8_Br_8_; (c) BA_2_MA_4_Pb_6_Br_16_; (d) The trapped electron density dependence wavelength curves at a cross-sections 10 nm near Al electrode.

**Figure S9.** The tDOS of quasi-2D perovskite-organic PM-NPDs based on BA2MA4Pb5I16 (I-rich), BA2MA4Pb5I8Br8 (Br-50%), and BA2MA4Pb5Br16 (Br-rich).


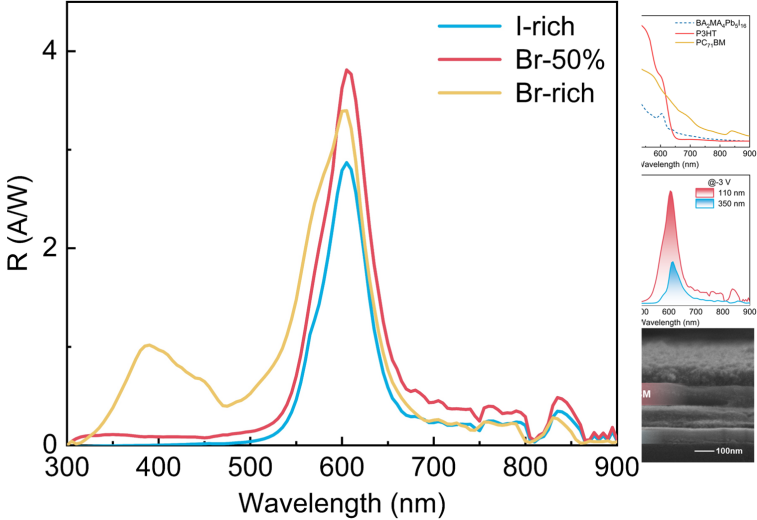


**Figure S10.** The responsivity spectrum based various perovskite filter layers.

**
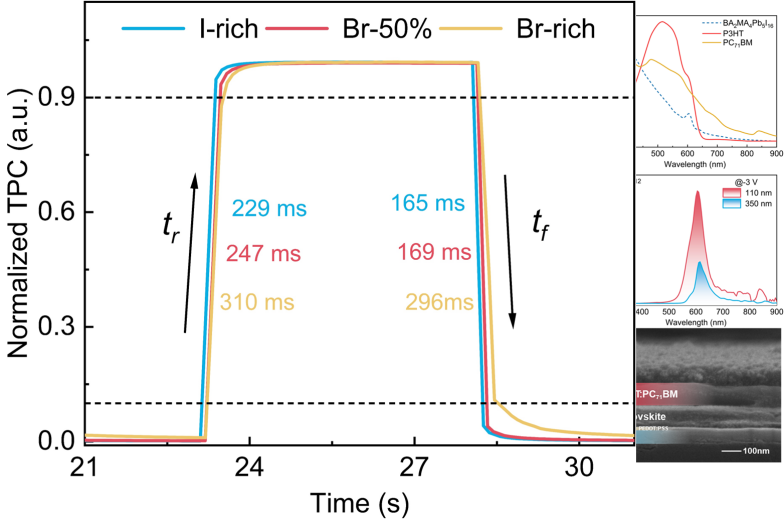
**

**Figure S11.** The normalized TPC based various perovskite filter layers.


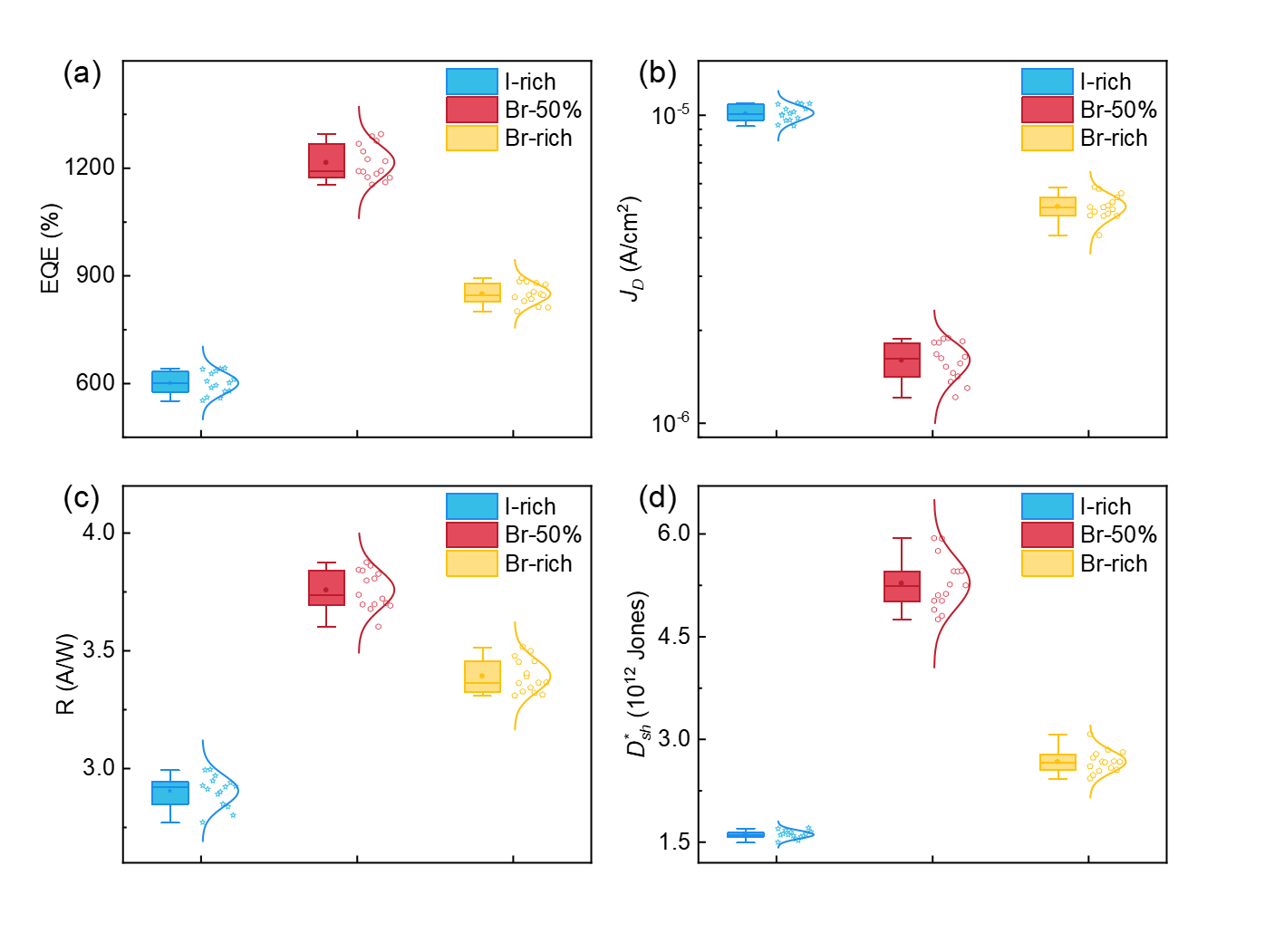


**Figure S12.** The box plots and normal distribution curve of the key performance parameters for 15 independent PM-NPDs based various perovskite filter layers: (a) EQE; (b) *J_D_*; (c) R; (d) $D_{sh}^{*}$.

## References

[1] J. Jiao, Y. Zhang, L. Shi, et al.,"High Responsivity of Narrowband Photomultiplication Organic Photodetector via Interfacial Modification," *Advanced Optical Materials*  11 no. 12 (2023): 2203132, https://doi.org/10.1002/adom.202203132.

[2] L. C. Winkler, J. Kublitski, J. Benduhn, et al.,"Photomultiplication Enabling High‐Performance Narrowband Near‐Infrared Organic Photodetectors," *Advanced Electronic Materials*  9 no. 9 (2023): 2201350, https://doi.org/10.1002/aelm.202201350.

[3] Q. Zhang, Y. Feng, J. Hu, et al.,"Achieving Photomultiplication in Dye‐Sensitized Narrowband Photodetectors by Electron Injection through a Thin Hole‐Transporting Layer," *Advanced Optical Materials*  10 no. 7 (2022): 2102266, https://doi.org/10.1002/adom.202102266.

[4] G. Suthar, C. W. Chu, F. C. Chen,"High‐Performance Self‐Filtering Organic Photodetectors with Photomultiplication Narrowing," *Advanced Optical Materials*  12 no. 21 (2024): 2400662, https://doi.org/10.1002/adom.202400662.

[5] Z. Lan, Y. Lei, W. K. E. Chan, et al.,"Near-infrared and visible light dual-mode organic photodetectors," *Science Advanced* 6 no. 5 (2020): eaaw8065, https://doi.org/doi:10.1126/sciadv.aaw8065.

[6] Z. Zhao, C. Xu, Y. Ma, et al.,"Ultraviolet Narrowband Photomultiplication Type Organic Photodetectors with Fabry−Pérot Resonator Architecture," *Advanced Functional Materials*  32 no. 29 (2022): 2203606, https://doi.org/10.1002/adfm.202203606.

[7] X. Zhao, K. Yang, X. Ma, et al.,"Ultraviolet and visible dual-narrowband photomultiplication type organic photodetectors," *Chemical Engineering Journal*  517 (2025), https://doi.org/10.1016/j.cej.2025.164473.

[8] X. Zhao, H. Qu, Z. Zhuo, et al.,"Highly Sensitive Near‐Infrared Narrowband Photomultiplication Type Organic Photodetectors via Employing Charge Injection Narrowing and Exciton Dissociation Narrowing Strategy," *Advanced Functional Materials*  (2025): 2522181, https://doi.org/10.1002/adfm.202522181.
